# Cerebral venous sinus thrombosis as a complication of cranial melioidosis – a rare case report

**DOI:** 10.1099/acmi.0.000357

**Published:** 2022-05-27

**Authors:** Biji Bahuleyan, Manuel Adarsh, Jayachandran Akarsh, Arun Kumar M. L., Chandra S. Rohitha, George Xavier Elenjickal, Sreevalsan T. V., Santhosh George Thomas

**Affiliations:** ^1^​ Department of Neurosurgery, Lisie Hospital, Ernakulam, Kerala, India; ^2^​ Department of Neurology, Lisie Hospital, Ernakulam, Kerala, India; ^3^​ Department of Microbiology, Lisie Hospital, Ernakulam, Kerala, India; ^4^​ Department of Internal Medicine, Lisie Hospital, Ernakulam, Kerala, India; ^5^​ Department of Critical care Medicine, Lisie Hospital, Ernakulam, Kerala, India

**Keywords:** melioidosis, cerebral abscess, cerebral venous sinus thrombosis, *Burkholderia pseudomallei*, osteomyelitis

## Abstract

Cerebral venous sinus thrombosis is a rare complication of cranial melioidosis. We report a case of an adult male who presented with skull osteomyelitis, transverse sinus thrombosis and multiple brain abscesses. His blood cultures grew *Burkholderia pseudomallei *. The patient finally succumbed after multiple recurrences of the infection despite surgical excision of the osteomyelitic bone and the recommended antibiotic treatment. The management of cerebral venous sinus thrombosis in patients with cranial melioidosis is discussed along with a brief review of the literature.

## Introduction

Melioidosis is an infection caused by the soil-dwelling Gram-negative organism, *Burkholderia pseudomallei* [1, 2]. Infection of the central nervous system (CNS) by this organism, known as cranial melioidosis (CM) is rare [1, 3]. CM can involve any structure in and around the CNS [1, 2, 4]. The case we present is novel in that simultaneous occurrence of skull osteomyelitis, cerebral venous sinus thrombosis (CVT), multiple cerebral abscesses and systemic melioidosis in the same patient, is a clinical entity that has not been reported so far. Our case is also the fifth reported case of CVT in a patient with CM.

## Clinical presentation

This 51-year-old male patient with diabetes mellitus (DM) was admitted six times at our centre for treatment, the details of which are summarized in Table 1. He had multiple admissions as there was recurrence of infection despite recommended treatment during each of these admissions.

**Table 1.** Details of the multiple hospital admissions

Admission	History and examination	Brain imaging	Blood investigations	Treatment	Course
First	Fever, HA, retro mastoid swelling	CT: Erosion of skull near Lt TS and adjacent soft tissue swelling (Fig. 1a) MRI and MRV: Contrast enhancement involving the dura, occipital bone and overlying scalp (Fig. 1b), Lt TS thrombosis (Fig. 1c)	Blood C/S : negative for AFB, fungal and routine. CSF study: normal	Empirical: IV VAN, CFP-TAZ x 3 wks f/b IV CZM x 2 wks (as renal parameters worsened) f/b P/O CIP x 2 wks (Total 7 wks) Denied surgical excision	Improved
Second (3 wks after d/d)	Fever, retro mastoid swelling	MRI: Same as first admission	Blood C/S: *Pseudomonas aeruginosa*sensitive PIP-TAZ, LEV, MEP, CZM, CFP, CIP	PIP-TAZ x 2 wks Denied surgical excision	Improved
Third (4 wks after d/d)	Altered sensorium	MRI: Extension of the bony and soft tissue swelling	Blood C/S: *B. pseudomallei* sensitive to CZM, MEP, MIN, TMP-SMX Histopathology: Chronic osteomyelitis	Sx :Excision of the osteomyelitic occipital bone. IV CZM (renal adjusted dose) x 3 wks f/b MEP (renal adjusted dose) x 5 wks. P/O TMP-SMX and MIN x 24 wks	Improved
Fourth (36 wks after d/d)	Fever, Lt hemiparesis	MRI: Multiple contrast enhancing lesions involving both cerebral hemispheres, cerebellum and brain stem (Fig. 2a, b)	Blood and urineC/S: Sterile	IV CZM, MTG, VAN (renal adjusted dose) x 2 wks P/O MIN and LEV x 6 wks	Improved Resolution of brain abscesses on MRI
Fifth (12 wks after d/d)	Fever, GTCS, Lt hemiparesis	MRI: Right frontoparietal T2 weighted hyperintense lesion	Blood and urine C/S: Sterile	AMX-CLV x 2 wks	Worsened
Sixth (6 wks after d/d)	Lt hemiparesis	MRI: Right frontoparietal white matter contrast enhancing lesion (Fig. 2c)	Not done as they refused	MIN and LEV	Lost to FU Expired 8 wks after the last FU

*wks* weeks, *d/d* discharge, *GTCS* generalized tonic clonic seizure, *Lt* left, *HA* headache, *CT* computed tomography, *MRI* magnetic resonance imaging, *MRV* magnetic resonance venogram, *TS* transverse sinus, *AFB* acid fast bacilli, *CSF* cerebrospinal fluid, *VAN* vancomycin, *CFP* cefepime, *TAZ* tazobactam, *CZM* ceftazidime, *IV* intravenous, *P/O* per oral, *C/S* culture and sensitivity, *PIP-TAZ* piperacillin and tazobactam, *MEP* meropenem, *MIN* minocycline, *TMP-SMX* co-trimoxazole, *MTG* metrogyl, *CIP* ciprofloxacin, *LEV* levofloxacin, *AMX-CLV* amoxicillin and clavulanic acid, *FU* follow up, *Sx* surgery, *f/b* followed by.

**Fig. 1.**
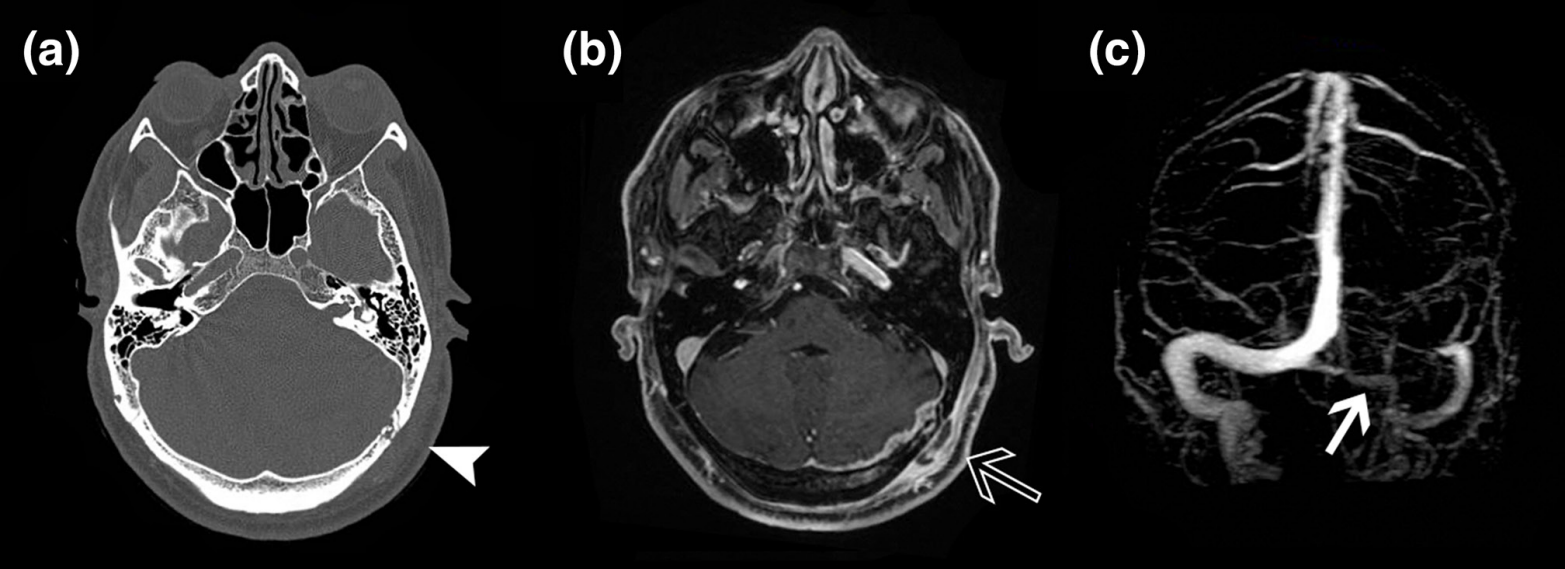
(a) The axial view of CT bone window of the skull showing bony erosion near the left TS and adjacent soft tissue swelling (white arrowhead). (b) The axial view of contrast MRI scan of the brain showing enhancement involving the dura, occipital bone and overlying scalp (empty arrow). (c) The coronal MIP reformation of MR venogram of the brain showing Lt TS thrombosis (white arrow).

**Fig. 2.**
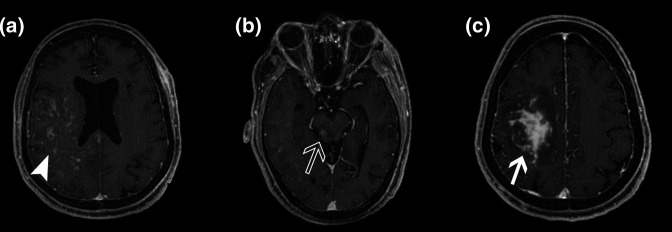
(a) The axial view of contrast MRI scan of the brain showing multiple contrast enhancing lesions involving the right cerebral hemisphere (white arrowhead). (b) The axial view of contrast MRI scan of the brain showing contrast enhancing lesion in the brainstem (empty arrow). (c) The axial view of contrast MRI scan of the brain showing right frontoparietal white matter contrast enhancing lesion (white arrow).

In the first admission, contrast computed tomogram (CT) of the brain showed imaging features suggestive of focal osteomyelitis of the left occipital bone near the transverse sinus (Fig. 1a). Magnetic resonance imaging (MRI) of the brain showed focal pachymeningitis in the left occipital region (Fig. 1b). Magnetic resonance venogram showed left transverse sinus (TS) thrombosis (Fig. 1c). Blood and cerebrospinal fluid (CSF) cultures were sterile. Excision, biopsy and culture of the occipital bony lesion was planned. Patient and relatives were unwilling for any surgical procedure. Hence he was started on empirical intravenous antibiotics followed by oral antibiotics for a total duration of 7 weeks along with antiepileptic medications and blood thinners. He became afebrile and the retromastoid swelling subsided.

During the second admission, blood culture grew *Pseudomonas aeruginosa *. He was treated with culture specific intravenous antibiotics for 2 weeks with which he improved clinically.

He presented in a state of altered sensorium during the third admission. MRI of the brain showed progression of the bony erosion. He underwent left retromoastoid craniectomy and excision of the osteomyelitic occipital bone. The histopathology of the bone specimen was suggestive of chronic osteomyelitis. Blood culture grew *B. pseudomallei *. He was started on culture-specific intravenous and oral antibiotics simultaneously. Intravenous antibiotics were given for 8 weeks and the oral antibiotics were continued for a total of 24 weeks. He remained asymptomatic for about 36 weeks from the time of discharge.

During the fourth admission, he presented with left hemiparesis. MRI of the brain showed multiple contrast enhancing ill-defined lesions involving grey and white matter of both cerebral hemispheres, cerebellum and brain stem (Fig. 2a, b). Blood and urine cultures were sterile. He was treated with empirical intravenous followed by oral antibiotics based on the previous positive *B. pseudomallei* culture sensitivity for a total of 6 weeks. His general condition and hemiparesis improved.

During the fifth and sixth admissions, the MRI of the brain showed progression of the ill-defined lesions with development of a large enhancing lesion in the right frontoparietal white matter (Fig. 2c). Blood and urine cultures remained sterile. He was treated with empirical antibiotics but lost to follow up and subsequently expired.

## Discussion

Melioidosis is endemic in Southeast Asia and Northern Australia [3]. Infection with this organism occurs via ingestion, inhalation or percutaneous inoculation [2, 3]. CNS infection is believed to be secondary to hematogenous spread or direct spread from the nasopharynx [2].

Melioidosis can affect any organ of the body and it can cause blood-stream infections (septicemia) as seen in our case. The organism is known to cause scalp abscess [4], pachymeningitis [4], skull osteomyelitis [1], cerebral abscesses [1], cranial subdural empyema [2], meningoencephalitis [5], meningitis [4], encephalomyelitis [2] or CVT [4, 6–8]. Encephalomyelitis is the presentation with the worst prognosis [2]. When melioidosis involves the spinal cord a wrong diagnosis of demyelination can often lead to the initiation of steroids, which can be detrimental [2]. In reported cases of CM with CVT the CNS involvement includes venous infarct [8], cerebral abscess [6, 7], cerebellar abscess [6], subdural collection [7], pachymeningeal thickening [4] and skull osteomyelitis [7].

Involvement of multiple tissue layers from the scalp to the brain parenchyma as reported by Pit *et al*. was seen in our case [9].

The previously reported four cases of CM with CVT and the case we present are summarized in Table 2. Niyasom *et al*. reported the first case of CM with CVT [8]. Patients with CM with CVT can have associated venous infarction in the cerebral hemispheres [8]. All patients were males with ages ranging from 23 to 69 [4, 6–8]. The commonest co-morbidity associated was DM [6, 8, 10]. The other risk factors include renal disease, thalassemia, previous trauma or surgery, pulmonary tuberculosis and malignancies [10]. Our patient had both DM and renal failure as co-morbidities. The venous sinus commonly involved was the superior sagittal sinus [6–8]. Two previously reported cases [4, 8] showed TS involvement as seen in our case.

**Table 2.** Cases of CM associated with CVT [4, 6–8]

Author	Age/sex	Co-morbidities	CVT location	Other CNS involvement	Treatment	Outcome (FU wks)
Niyasom *et al*. [8]	42 /M	DM, liver cirrhosis	SSS,TS, SS	Parietal lobe infarct	CZ x 2 wks	Improved (2 wks)
Nayak *et al*. [4]	23 /M	Nil	TS, SS, jugular vein	Pachymeningeal thickening temporal region and petrous apex	Biopsy f/b IV CZ x 6 wks f/b P/O TMP-SMX x 24 wks	Improved (24 wks)
Abeysundara *et al*. [6]	69 /M	DM	SSS	Multiple cerebral and cerebellar abscesses	Empirical: IV MP and VM x 4 wks Definitive: IV MP and P/O TMP-SMX x 2 wks f/b P/O DC and P/O TMP-SMX x 20 wks	Improved (20 wks)
Muthusamy *et al*. [7]	33 /M	Nil	SSS	Cerebral abscess, subdural collection and skull osteomyelitis	Craniectomy and drainage of the abscess f/b antibiotic treatment (NA)	Improved (52 wks)
Our case	51 /M	DM Renal failure	TS	Multiple brain abscesses, skull osteomyelitis	Excision of the osteomyelitic occipital bone and antibiotic treatment as detailed in Table 1	Multiple recurrences

*M* Male, *DM* diabetes mellitus, *CNS* central nervous system, *CVT* cerebral venous sinus thrombosis, *SSS* superior sagittal sinus, *TS* transverse sinus, *SS* sigmoid sinus, *CZ* ceftazidime, TMP-SMX co-trimoxazole, *MP* meropenem, *VM* vancomycin, *DC* doxycycline, *IV* intravenous, *P/O* per oral, *f/b* followed by, *FU* follow up, *wks* weeks, *na* data not available.

Delay in diagnosis is a major concern in patients with melioidosis [1]. The clinical and radiological features of CM can mimic other CNS pathologies like tuberculosis [1, 4, 11], other pyogenic infections [11] or malignancies [1]. In our patient also there was a delay in diagnosis as the organism could not be isolated in the first two admissions. This made us treat him for a shorter duration than the recommended prolonged antibiotic treatment. However, after the recommended culturespecific treatment he was disease free for about 9 months.

Cultures from the CNS specimen is the best way to establish a definite diagnosis of CM [12]. Diagnosis of the disease can also be done by culture of pus from the abscess [7], blood [6, 8] or involved tissues [4] demonstrating the growth of the organism. CSF pictures can be variable in patients with CM. It can show lymphocytic pleocytosis [2, 12], a CSF picture similar to tuberculosis or viral encephalitis. In about a third of patients, polymorphonuclear cell predominance can be seen [12] and rarely it can be acellular [6, 8]. CSF also demonstrates high protein and normal glucose [12]. However, in our case, the CSF study was normal and CSF culture was negative.

The appropriate surgical intervention followed by antibiotic therapy is the recommended treatment for CM. The antibiotic of choice and its optimal duration of treatment are controversial [13]. Various authors recommend intravenous followed by oral antibiotic therapy in melioidosis. Duration of parenteral antibiotic therapy recommended by various authors ranges from 2 to 6 weeks [1–3] followed by oral antibiotics for 3–6 months [1, 3]. The parenteral antibiotics used are ceftazidime [2, 3] or meropenem [2]. Maintenance therapy with oral co-trimoxazole for 6 months following the initial parenteral antibiotic treatment has been recommended [1, 2, 4]. In patients who cannot tolerate co-trimoxazole, amoxicillin-clavulanate and/or doxycycline can be administered [3, 4]. The choice of antibiotic should be based on the culture and sensitivity. CVT in these patients is treated with intravenous anticoagulants [4, 8] along with antiepileptics [8].

Of the four previously reported cases of CM with CVT, all had good outcomes [4, 6–8]. When CM is seen associated with osteomyelitis or mass lesion, the mortality rate was about 25 % [1]. CM is known to recur due to non-compliance or suboptimal duration of antibiotic treatment [1]. We feel that the reasons for multiple recurrences and death in our case are (i) delay in diagnosis, (ii) inappropriate treatment due to lack of isolation of the organism during the initial two admissions, (iii) presence of co-morbidities like DM and renal failure and (iv) noncompliance to treatment.

## Conclusion

Clinicians should be aware of this rare presentation of simultaneous CNS and systemic infection in association with CVT in patients with melioidosis.

Through this article, we recommend the following in patients with clinical presentation similar to ours (i) consider CM as one of the differential diagnoses in patients presenting with intracranial lesion, (ii) consider performing MR venogram in these patients as CVT can rarely be associated, (iii) consider long-term recommended antibiotic therapy and (iv) keep these patients on close clinical and radiological follow-up. If cranial imaging shows recurrence of infection and bacterial cultures remain negative, these patients should be treated again with antibiotics based on previous positive cultures for the recommended period of time.
